# Increased mitophagy in the skeletal muscle of spinal and bulbar muscular atrophy patients

**DOI:** 10.1093/hmg/ddx019

**Published:** 2017-01-13

**Authors:** Doriana Borgia, Adriana Malena, Marco Spinazzi, Maria Andrea Desbats, Leonardo Salviati, Aaron P. Russell, Giovanni Miotto, Laura Tosatto, Elena Pegoraro, Gianni Sorarù, Maria Pennuto, Lodovica Vergani

**Affiliations:** 1 Department of Neurosciences, University of Padova, Padova, Italy; 2 VIB Center for the Biology of Disease, KU Leuven Center for Human Genetics, Leuven, Belgium; 3 Clinical Genetics Unit, Department of Woman and Child Health, University of Padova, Padova, Italy, and IRP Città della Speranza, Padova, Italy; 4 Institute for Physical Activity and Nutrition, School of Exercise and Nutrition Sciences, Deakin University, Burwood, Australia; 5 Department of Molecular Medicine, University of Padova, Padova, Italy; 6 Proteomic Center of Padova University, VIMM and Padova University Hospital, Padova, Italy; 7 Dulbecco Telethon Institute, Centre for Integrative Biology, University of Trento, Trento, Italy

## Abstract

Spinal and bulbar muscular atrophy (SBMA) is a neuromuscular disorder caused by polyglutamine expansion in the androgen receptor (AR) and characterized by the loss of lower motor neurons. Here we investigated pathological processes occurring in muscle biopsy specimens derived from SBMA patients and, as controls, age-matched healthy subjects and patients suffering from amyotrophic lateral sclerosis (ALS) and neurogenic atrophy. We detected atrophic fibers in the muscle of SBMA, ALS and neurogenic atrophy patients. In addition, SBMA muscle was characterized by the presence of a large number of hypertrophic fibers, with oxidative fibers having a larger size compared with glycolytic fibers. Polyglutamine-expanded AR expression was decreased in whole muscle, yet enriched in the nucleus, and localized to mitochondria. Ultrastructural analysis revealed myofibrillar disorganization and streaming in zones lacking mitochondria and degenerating mitochondria. Using molecular (mtDNA copy number), biochemical (citrate synthase and respiratory chain enzymes) and morphological (dark blue area in nicotinamide adenine dinucleotide-stained muscle cross-sections) analyses, we found a depletion of the mitochondria associated with enhanced mitophagy. Mass spectrometry analysis revealed an increase of phosphatidylethanolamines and phosphatidylserines in mitochondria isolated from SBMA muscles, as well as a 50% depletion of cardiolipin associated with decreased expression of the *cardiolipin synthase* gene. These observations suggest a causative link between nuclear polyglutamine-expanded AR accumulation, depletion of mitochondrial mass, increased mitophagy and altered mitochondrial membrane composition in SBMA muscle patients. Given the central role of mitochondria in cell bioenergetics, therapeutic approaches toward improving the mitochondrial network are worth considering to support SBMA patients.

## Introduction

Spinal and bulbar muscular atrophy (SBMA), also named Kennedy’s disease, is a neuromuscular disorder characterized by the late-onset and progressive loss of motor neurons from the brainstem and spinal cord, together with skeletal muscle weakness, fasciculations and atrophy (1). In addition to neuromuscular symptoms, SBMA patients develop endocrine and non-neuronal symptoms (2). SBMA is linked to CAG expansions in the exon 1 of the gene coding for the androgen receptor (AR) (3). In healthy subjects, this polyglutamine-encoding CAG trinucleotide tandem repeat has a length of no more than 36 repeats, and expansions over 38 cause disease. SBMA belongs to the family of polyglutamine diseases, which also includes Huntington’s disease (HD), dentatorubral-pallidoluysian atrophy (DRPLA) and spinocerebellar ataxia (SCA) type 1, 2, 3, 6, 7 and 17 (reviewed by 4). SBMA is a sex-specific disease with full manifestations restricted to males. The sex specificity of SBMA is owing to the higher serum levels of androgens in males compared with females (5), and is also observed in transgenic and knock-in mice expressing the polyglutamine-expanded AR (5–8). Moreover, a strikingly androgen-dependent phenotype manifests in transgenic flies expressing the polyglutamine-expanded AR, which develop signs of neurodegeneration only if fed a food containing androgens (9–11). Consistent with the androgen-dependent nature of SBMA, surgical and pharmacological castration of transgenic mice reduced disease manifestations, whereas treatment of female transgenic mice with androgens induced neurodegeneration (6,7,12). Although treatment of SBMA patients with anti-androgens exerts some beneficial effects in clinical trials (13,14), the applicability of this approach is limited by intrinsic side-effects.

Emerging evidence supports a key role for skeletal muscle in SBMA pathogenesis (15). Expression of polyglutamine-expanded AR in muscle is necessary and sufficient to cause disease. Indeed, the expression of polyglutamine-expanded AR in all tissues, except for skeletal muscle, prevented disease manifestations in transgenic mice (16). Moreover, knock-down of polyglutamine-expanded AR in peripheral tissues, including skeletal muscle, ameliorated the phenotype of SBMA mice (17). On the other hand, overexpression of non-expanded AR solely in muscle resulted in a phenotype reminiscent of SBMA (18). Analysis of muscle pathology in SBMA patients revealed the presence of neurogenic atrophy, such as angulated atrophic fibers and fiber-type grouping, and signs of myopathy, including fiber splitting, increased presence of central nuclei, and degeneration of fibers (19,20). In addition, SBMA muscles are characterized by the presence of hypertrophic fibers (20), a feature that is observed also in the skeletal muscle of transgenic SBMA mice at late stage of disease (21). In SBMA knock-in mice, muscles composed of both glycolytic and oxidative fibers, such as the quadriceps, are more severely affected then muscles mainly composed of oxidative fibers. Muscles composed of glycolytic and oxidative fibers undergo a metabolic switch toward an oxidative phosphorylation, a metabolic alteration that precedes denervation and is likely to result from intrinsic pathogenic processes occurring in these muscles (22). These metabolic alterations in knock-in SBMA mice were associated with mitochondrial abnormalities, induction of protein turnover, activation of mechanistic target of rapamycin (mTOR) signaling, and induction of autophagy at late stages of disease. While analysis of skeletal muscle in animal models of SBMA has been extensively reported, analysis of muscle pathology in SBMA patients is missing. Using skeletal muscle biopsies derived from SBMA patients, we investigated pathological processes underlying muscle weakness and wasting in SBMA patients. We found that polyglutamine-expanded AR accumulated in the nucleus and mitochondria of mature myotubes. Moreover, SBMA muscles were characterized by a reduction in mitochondrial mass, aberrant mitochondrial morphology, degenerating mitochondria with either a dense matrix or dilated-hypodense matrix and swelling, decreased mtDNA and citrate synthase (CS) activity, and enhanced autophagy and mitophagy. Finally, SBMA muscle mitochondria presented with altered mitochondrial membrane lipid composition and downregulation of *cardiolipin synthase* expression.

## Results

### Increased atrophy and hypertrophy indexes in the quadriceps muscle of SBMA patients

**Figure 1:**
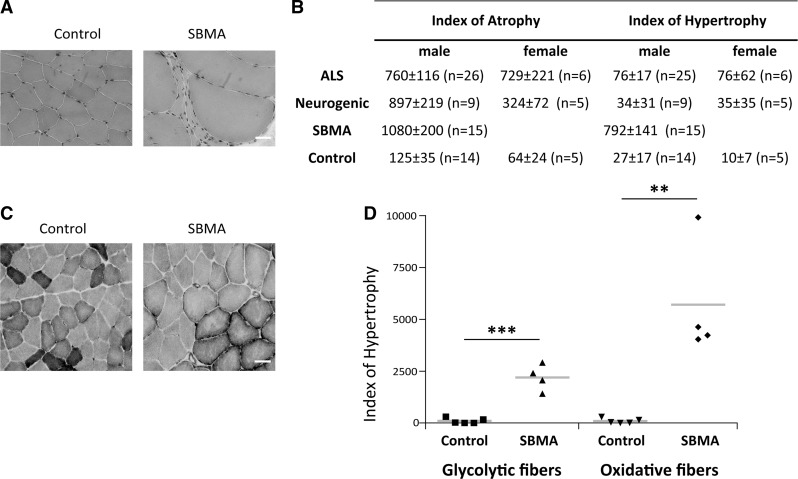
Atrophic and hypertrophic fibers in the skeletal muscle of SBMA patients. (**A**) Representative images of H&E-stained cryosections of control and SBMA quadriceps muscles. Scale bar, 40 µm. (**B**) Table showing the mean values of the atrophy and hypertrophy indexes in SBMA, ALS, and neurogenic patients and control subjects. The values, quantified as described in Materials and Methods, are expressed as mean ± SEM. Significance by ANOVA + LDS Fisher post doc: atrophy index: *P* < 0.001 SBMA, ALS and neurogenic atrophy patients versus controls; hypertrophy index: *P* < 0.001 SBMA versus controls, *P* < 0.001 SBMA versus ALS patients, *P* < 0.001 SBMA versus neurogenic patients. (**C**) Representative NADH-stained quadriceps muscle sections of control and SBMA subjects. Scale bar, 80 μm. (**D**) Analysis of the hypertrophy index in oxidative and glycolytic fibers. Graph, mean ± SEM, *n* = 4 SBMA patients and 5 control subjects. Significance by Student’s *t* test: **P < 0.01, ***P < 0.001.

To investigate in detail pathological processes occurring in the muscle of SBMA patients, we collected biopsy specimens from the quadriceps femoris of SBMA patients with 41–49 CAG repeats and age- and gender-matched control subjects (Supplementary Material, Table S1). Using hematoxylin and eosin (H&E) analysis, we detected signs of neurogenic atrophy together with myopathic changes, such as fiber splitting and increased internal nuclei (Fig. 1A;Supplementary Material, S1), as others and we have previously described in SBMA patients (19,20) and mice (21,22). In addition, we detected several hypertrophic fibers, as previously reported (20). Here we sought to analyze in detail both the atrophic and hypertrophic fibers present in SBMA muscle. Male muscle fiber size ranges between 40 and 80 μm, and the size of atrophic and hypertrophic fibers is <40 and >80 μm, respectively (23). We measured the fiber atrophy and hypertrophy indexes (HIs) in the muscle of SBMA patients as described in the Material and Methods section (Fig. 1B). As controls, we compared the muscle pathology of SBMA patients with that of patients suffering from either amyotrophic lateral sclerosis (ALS) or other neurogenic diseases. We found that the atrophy index (AI) was significantly (*P* < 0.001) increased by >6-fold in the muscle of SBMA, ALS and neurogenic patients compared with control subjects. On the other hand, the HI was significantly (*P* < 0.001) increased by 29-fold in SBMA patients, whereas it was slightly and not significantly increased by <3-fold in both ALS and neurogenic patients, indicating that a high HI is a feature of the muscle pathology of SBMA patients. Glycolytic fibers are more severely affected than oxidative fibers in muscles composed of mixed fibers, such as quadriceps, in knock-in SBMA mice (22). Using nicotinamide adenine dinucleotide (NADH) staining, we measured the HI of oxidative and glycolytic fibers in the quadriceps muscle of SBMA patients and control subjects (Fig. 1C and D;Supplementary Material, S2). The HI of glycolytic and oxidative fibers was increased by 22- and 57-fold, respectively, indicating that hypertrophy of the oxidative fibers exceeds that of glycolytic fibers in the muscles of SBMA patients. These results indicate that the presence of hypertrophic fibers is a key feature of SBMA muscle, with oxidative fibers having a higher HI compared with glycolytic fibers.

### Aberrant accumulation of polyglutamine-expanded AR in the nucleus and mitochondria of SBMA muscle

**Figure 2:**
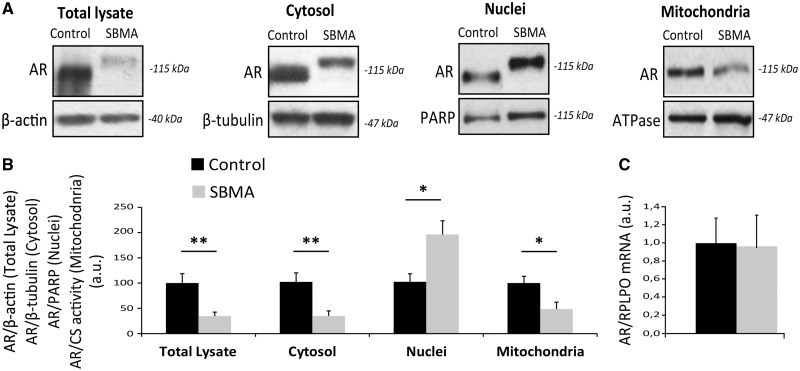
Aberrant subcellular localization of polyglutamine-expanded AR in the muscle of SBMA patients. (**A**) Western blotting analysis of AR levels in total lysates, cytosolic, nuclear and mitochondrial fractions from quadriceps muscles of SBMA patients and control subjects. β-actin, β-tubulin, PARP and ATPase were used as loading controls. (**B**) Quantification of AR in total lysates, and nuclear, cytosolic and mitochondrial fractions. Graph, mean ± SEM, *n* = 7 SBMA patients and 7 control subjects. Poly(ADP-ribose) polymerase (PARP), citrate synthase (CS). Significance by Student’s *t* test: *P < 0.05, **P < 0.01. (**C**) Real-time PCR analysis of the AR mRNA transcript levels normalized to *large ribosomal protein* (*RPLPO*) mRNA in the muscle of SBMA patients and control individuals. Graph, mean ± SEM, *n* = 6 SBMA patients and 3 control subjects. Significance by Student’s *t* test: *P < 0.05, **P < 0.01.

Next, we analyzed AR expression levels in the muscle of SBMA patients and control subjects (Fig. 2A and B). AR levels were significantly reduced by 65% in the quadriceps muscle of SBMA patients, despite no significant changes in the AR transcript levels (Fig. 2C). We have previously shown that polyglutamine-expanded AR is enriched in the nucleus of cultured myotubes derived from the muscle of SBMA patients (24), and it localizes to mitochondria (25). Therefore, we analyzed the accumulation of AR in the cytosolic, nuclear and mitochondrial fractions of SBMA muscle tissues and age-matched control samples. As positive control, we verified the enrichment of the nuclear marker, poly(ADP-ribose) polymerase (PARP), in the nuclear fraction (Supplementary Material, Fig. S3). To assess mitochondrial fraction purity we verified that our mitochondrial fraction did not contain β-tubulin and was enriched with the mitochondrial import receptor subunit TOM20 (Supplementary Material, Fig. S4). Importantly, accumulation of polyglutamine-expanded AR was decreased by 66% in the cytosolic fraction and increased by 2-fold in the nuclear fraction compared with normal AR (Fig. 2A and B). Moreover, AR signal localized to mitochondria was decreased by 52% in SBMA muscles compared with control muscles. These results indicate that accumulation of total polyglutamine-expanded AR is decreased in the cytosol and mitochondria, but enriched in the nucleus in the muscle of SBMA patients.

### Reduced number and altered morphology of mitochondria in the muscles of SBMA patients

**Figure 3:**
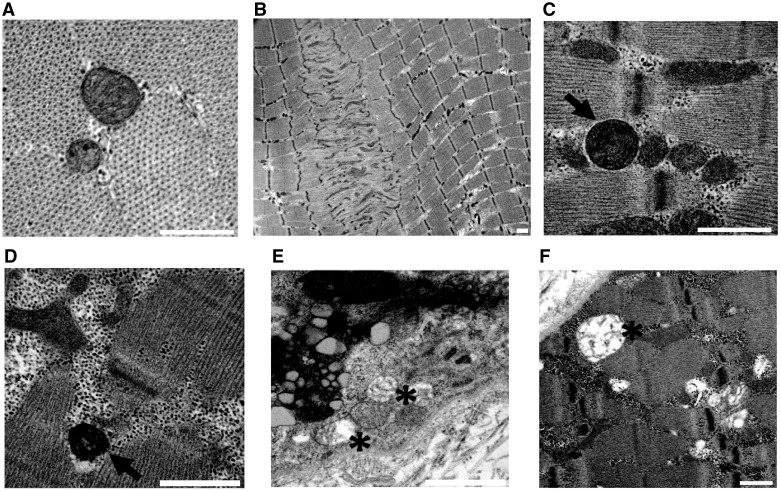
Mitochondrial abnormalities in the muscle of SBMA patients. (**A**) Representative image of TEM analysis of control muscle specimens. Representative images of TEM analysis of SBMA muscle specimens. (**B**) TEM analysis revealed myofibrillar disorganization and Z-line streaming. (**C**, **D**) Degenerated mitochondria with dense matrix (arrows). (**E**, **F**) Mitochondria with dilated-hypodense matrix and swelling (asterisks). Scale bar, 1 μm.

**Figure 4:**
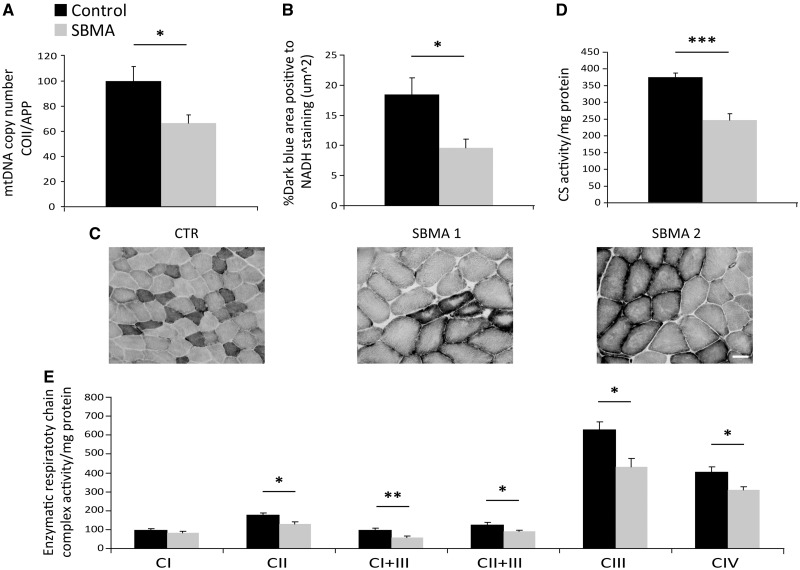
Reduction of mitochondria in the muscle of SBMA patients. (**A**) Real-time PCR analysis of mtDNA copy number measured as the ratio between *cytochrome c oxidase II* (*COII*) and nuclear *amyloid precursor protein* (*APP*) genes. Graph, mean ± SEM, *n* = 13 SBMA patients and 14 control subjects. (**B**) Mitochondrial mass expressed as the percentage of dark blue area in NADH-stained muscle cross-sections. Graph, mean ± SEM, *n* = 6 SBMA patients and 5 control subjects. (**C**) NADH analysis of quadriceps muscle of control and SBMA subjects. Scale bar, 80 μm. (**D**) Citrate synthase (CS) activity, expressed as nmol min^−1^ mg^−1^ of protein. Graph, mean ± SEM, *n* = 6 SBMA patients and 4 control subjects. (**E**) Activity of the respiratory chain complexes I–IV, expressed as nmol min^−1^ mg^−1^ of protein. Graph, mean ± SEM, *n* = 6 SBMA patients and 4 control subjects. Significance by Student’s *t* test: *P < 0.05, **P < 0.01, ***P < 0.001.

To investigate whether mitochondrial morphology and localization was altered in the muscle of SBMA patients, we performed transmission electron microscopy (TEM) on quadriceps femoris. Ultrastructural examination of muscle tissues from control individuals (Fig. 3A) and SBMA patients (Fig. 3B–F) revealed myofibrillar disorganization and streaming in zones lacking mitochondria (Fig. 3B) and degenerating mitochondria with either a dense matrix (Fig. 3C and D) or dilated-hypodense matrix (Fig. 3E) and swelling (Fig. 3F). Next, we assessed mitochondrial abundance by molecular, biochemical and morphological analyses on SBMA and control muscles. Using real-time PCR, we found that mitochondrial DNA (mtDNA) copy number was significantly reduced by 40% in the muscle of SBMA patients compared with control subjects (Fig. 4A). Using NADH staining, we noticed the presence of moth-eaten fibers with oxidative enzyme activity progressively reduced from the periphery to the center of the fibers, suggesting alterations in the mitochondrial distribution and activity in SBMA oxidative fibers (Figs 1C, 4B and C;Supplementary Material, S2). Mitochondrial mass was also assessed by measuring the percentage of dark blue (NADH-positive) area for field using ImageJ software (see Materials and Methods). This analysis demonstrated a 48% reduction in mitochondrial amount in SBMA muscle compared with controls (Fig. 4B and C). The activity of the mitochondrial matrix enzyme, CS, was decreased by 35% in SBMA muscle (Fig. 4D). The enzymatic activity of respiratory chain complexes (OXPHOS) II, III, and IV was also significantly decreased by 24–40% in SBMA muscle (Fig. 4E). However, OXPHOS rates normalized to CS (Supplementary Material, Fig. S5A), and supercomplex expression and assembly (Supplementary Material, Fig. S5B) were normal in SBMA muscle. Altogether, these ultrastructural and biochemical analyses revealed altered mitochondrial distribution and reduced mitochondrial mass in SBMA muscle.

We then asked whether the reduction of mitochondrial amount in SBMA muscle was associated with altered expression of genes involved in mitochondrial biogenesis and function, such as peroxisome proliferator-activated receptor γ coactivator 1 alpha (PGC1*α*, *PPARGC1α*) and PGC-1β *(PPARGC1β*), which control muscle metabolism and mitochondrial biogenesis, mitochondrial transcription factor A *(TFAM)*, which regulates mitochondrial gene transcription and mitochondrial genome replication, estrogen-related receptor α (*ERR*α), which regulates the expression of nuclear genes involved in mitochondrial homeostasis and biogenesis, nuclear respiratory factor 1 (*NRF1*), a transcription factor that induces the expression of metabolic nuclear genes required for mitochondrial respiration, DNA transcription and replication and that has recently been linked to SBMA pathogenesis (26), cytochrome C oxidase subunit 4 *(COX4)*, Mn superoxide dismutase *(MnSOD*), a mitochondrial enzyme that protects mitochondria from oxidative damage, and mitofusin 1 and 2 *(MFN1* and *2*), which are involved in mitochondrial fusion (Supplementary Material, Fig. S6). In SBMA muscles all these genes were expressed similarly to control muscles. These data indicated that polyglutamine-expanded AR alters mitochondrial homeostasis without affecting the expression of genes related to mitochondrial biogenesis and function in skeletal muscle.

### Increased autophagy and mitophagy in the muscle of SBMA patients

**Figure 5:**
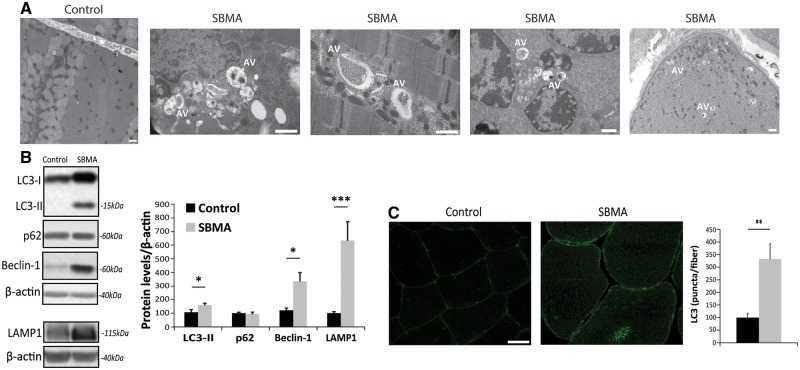
Increased autophagy in the muscle of SBMA patients. (**A**) TEM analysis of quadriceps muscle biopsies derived from two SBMA patients revealed the presence of autophagic vacuoles (AV). Scale bar, 1 μm. (**B**) Western blotting analysis of LC3-II, p62, Beclin-1 and LAMP1 levels. β-actin was used as loading control. Graph, mean ± SEM, *n* = 10 SBMA patients and 5 control subjects (top panels), and 9 SBMA patients and 9 control subjects (bottom panel). (**C**) Immunofluorescence analysis of LC3 in control and SBMA muscle tissues. The number of autophagosomes/fiber was measured as the number of green object-for-fiber (puncta/fiber). Graph, mean ± SEM, *n* = 50 fibers from each muscle sample derived from 11 SBMA patients and 10 control subjects. LC3 was detected using a specific antibody. Scale bar, 20 μm. Significance by Student’s *t* test: *P < 0.05, **P < 0.01, ***P < 0.001.

Reduction in the number of mitochondria can result from increased degradation, a process known as mitophagy, which normally occurs in cells to dispose damaged or superfluous mitochondria through selective autophagy (27–29). Mitophagy is a two-step process characterized by the induction of general autophagy followed by priming of the damaged mitochondria for mitophagic recognition and elimination (30). To determine whether mitophagy is induced in SBMA muscle, we first asked whether autophagy is enhanced in the muscle of SBMA patients. Ultrastructural examination of muscle tissues revealed the presence of autophagic vacuoles in SBMA muscle (Fig. 5A). Next, we analyzed the expression of autophagy markers, including the lipidated form of microtubule-associated protein 1A/1B-light chain 3 (LC3) II, which accumulates upon autophagosome formation, sequestosome 1 (*SQSTM1*, p62), which accumulates upon inhibition of autophagy flux, and Beclin-1 and lysosomal-associated membrane protein 1 (LAMP-1), which have previously been shown to be upregulated in the muscle of SBMA knock-in mice (22,31,32). We found that the expression levels of LC3II, Beclin-1, and LAMP1, but not p62, were significantly increased in the muscle of SBMA patients, indicating enhanced autophagy (Fig. 5B). To further corroborate the autophagic changes observed in SBMA muscle, we performed immunohistochemical analysis via confocal microscopy in muscle cryosections using an antibody against LC3 to mark autophagosomes. The LC3-positive puncta were quantified and expressed as puncta/myofiber (Fig. 5C). The accumulation of autophagic puncta was increased by 3-fold in SBMA muscles compared with control specimens. Notably, the LC3-positive puncta localized to the center of the fiber, which corresponds to the area with decreased NADH staining (Fig. 4C and Supplementary Material, S2). These observations indicate that autophagy is induced in the muscle of SBMA patients.

**Figure 6:**
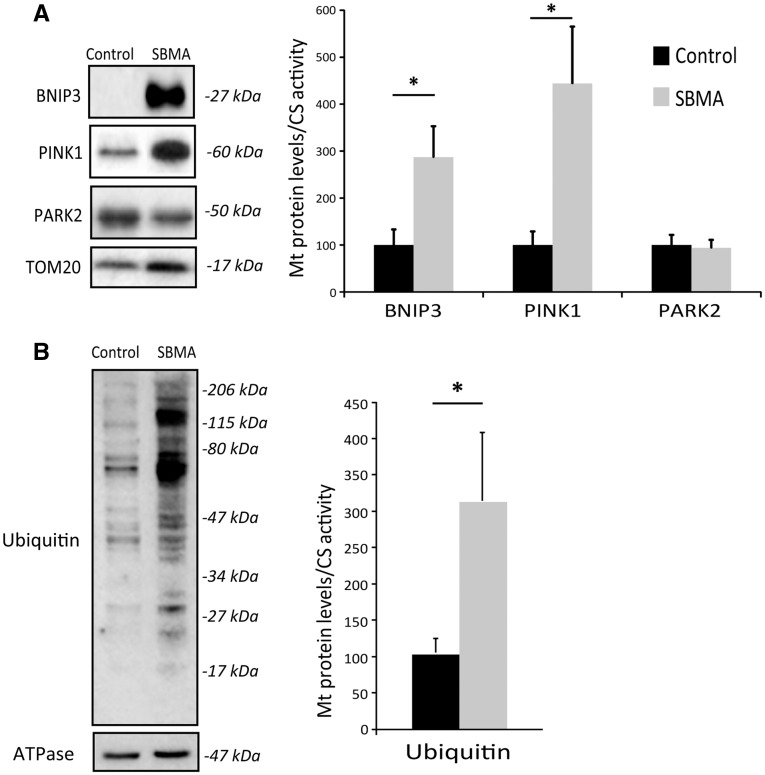
Mitophagy is induced in the muscle of SBMA patients. (**A**, **B**) Western blotting analysis of BNIP3, PINK1, PARK2 and ubiquitin levels in mitochondria isolated from the quadriceps muscle of SBMA patients and controls subjects normalized to CS activity. TOM20 and ATPase were used as loading controls. Graph, mean ± SEM, *n* = 5 SBMA patients and 5 control subjects. Significance by Student’s *t* test: *P < 0.05.

**Figure 7:**
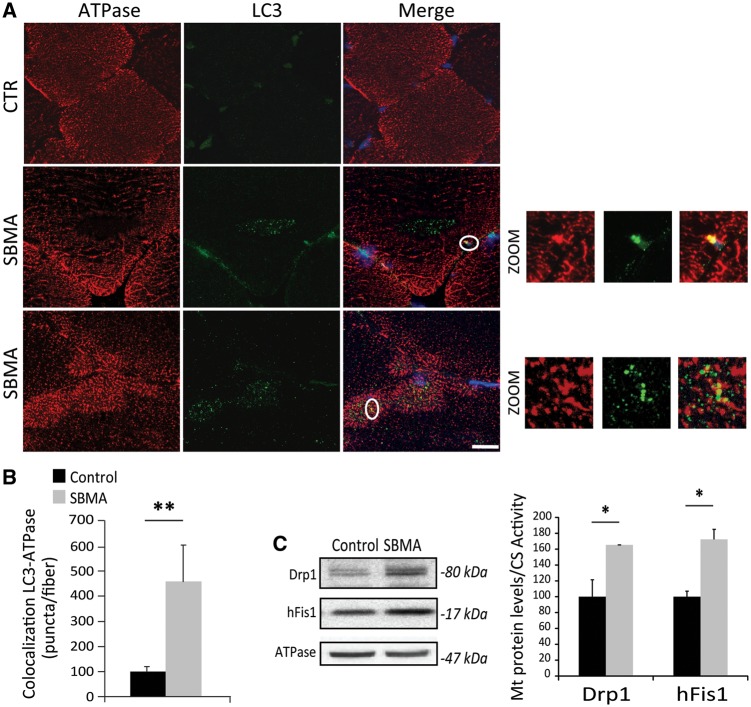
Increased mitophagy and mitochondrial fission proteins in the muscle of SBMA patients. (**A**) Representative images of anti-LC3 (green) and anti-ATPase (red) immunostaining of control and SBMA muscle tissues. Zoom: magnification of the marked area (circle). Scale bar, 20 μm. (**B**) Number of colocalized autophagosome-mitochondria (yellow puncta), measured as number of yellow puncta/fiber. Graph, mean ± SEM, *n* = 11 SBMA patients and 10 control subjects, *n* of fibers: 50 for each sample. (**C**) Western blotting analysis of Drp1 and hFis1 levels normalized to CS activity in mitochondria isolated from the quadriceps muscle of SBMA patients and control subjects. ATPase was used as loading control. Graph, mean ± SEM, *n* = 4 SBMA patients and 4 control subjects. Significance by Student’s *t* test: *P < 0.05, **P < 0.01.

Next, we assessed whether mitophagy is enhanced in the muscle of SBMA patients. To address this question, we isolated the mitochondrial fraction from SBMA and control muscles. We then analyzed the mitochondrial enrichment of proteins that prime damaged mitochondria and allow the recruitment of the autophagosome for mitophagy, such as BCL2/Adenovirus E1B 19kDa Interacting Protein 3 (BNIP3), PTEN-induced putative kinase 1 (PINK1), and the E3 ubiquitin-protein ligase parkin (PARK2) (29,33,34). BNIP3 and PINK1, but not PARK2, were increased by 3- and 4-fold, respectively, in isolated SBMA muscle mitochondria compared with controls (Fig. 6A). To test whether other E3 ubiquin ligases involved in mitophagy, such as MUL1 (35) and Gp78 (36), are induced in SBMA muscle, we measured the trascript levels of these factors by real-time PCR (Supplementary Material, Fig. S7). However, we did not find any increase in the expression of these genes in SBMA muscles compared with control specimens. Mitochondria degraded through mitophagy are polyubiquitinated (37). The amount of ubiquitinated proteins was augmented in the mitochondria isolated from SBMA muscles (Fig. 6B). By confocal microscopy analysis of quadriceps muscle cryosections, we found that the LC3-positive puncta colocalized with ATPase-positive-mitochondria, and double-positive vesicles were increased by 4.5-fold in SBMA muscle (Fig. 7A and B). Interestingly, we noticed that some SBMA muscle fibers had central regions rich in autophagic vacuoles (LC3-positive), but devoid of mitochondria, suggesting a mitophagic process that leads to removal of mitochondria. These areas overlapped with the NADH-negative central area of oxidative fibers (Fig. 4C;Supplementary Material, Fig. S2) and the myofibrillar disorganization and streaming in zones lacking mitochondria observed by TEM (Fig. 3B). Selective mitophagy is also controlled by mitochondrial dynamics (37). Mitochondrial fission facilitates mitophagy and is operated by specific mitochondria-associated proteins, such as dynamin-related protein 1 (Drp1) and mitochondrial fission 1 protein (hFis1) (27,38). Both Drp1 and hFis1 were significantly increased by 1.6- and 1.7-fold, respectively, in SBMA mitochondria, suggesting augmented fission events, in line with increased mitophagy (Fig. 7C). Taken together, these observations show that mitophagy is induced in the muscle of SBMA patients.

### Lipid composition of mitochondria is altered in the muscle of SBMA patients

**Figure 8:**
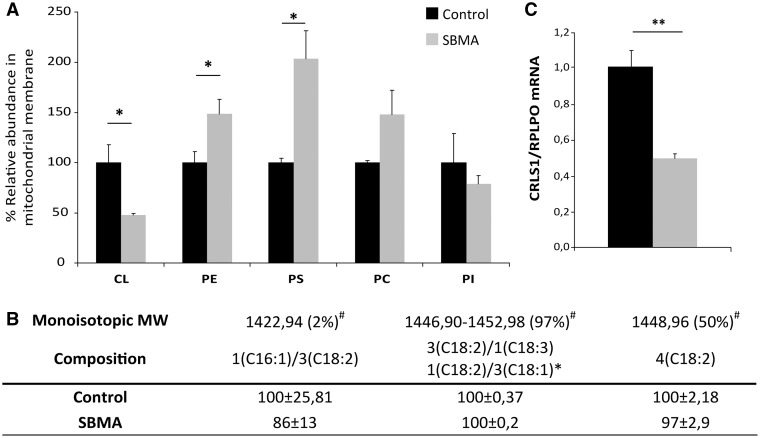
Reduced cardiolipin levels and biosynthesis in the muscle of SBMA patients. (**A**) Mass spectrometry lipidomic analysis of mitochondrial membranes isolated from SBMA patients and control subjects. Cardiolipin (CL), phosphatidylethanolamine (PE), phosphatidylserine (PS), phosphatidylcholine (PC) and phosphatidylinositol (PI) amounts were normalized to CS activity. Graph, mean ± SEM, *n* = 4 SBMA patients and 4 control subjects. (**B**) Composition of CL molecular species in control and SBMA muscle samples. The values are expressed as mean ± SEM of two independent experiments. ^#^Percentage of the molecular species/total CL. *Range of the major combinations among the shown acyl moieties. (**C**) Real-time PCR analysis of *cardiolipin synthase* (*CRLS1*) normalized to *RPLPO* in the muscle of SBMA patients and control subjects. Graph, mean ± SEM, n = 5 SBMA patients and 6 control subjects. Significance by Student’s *t* test: *P < 0.05, **P < 0.01.

**Figure 9:**
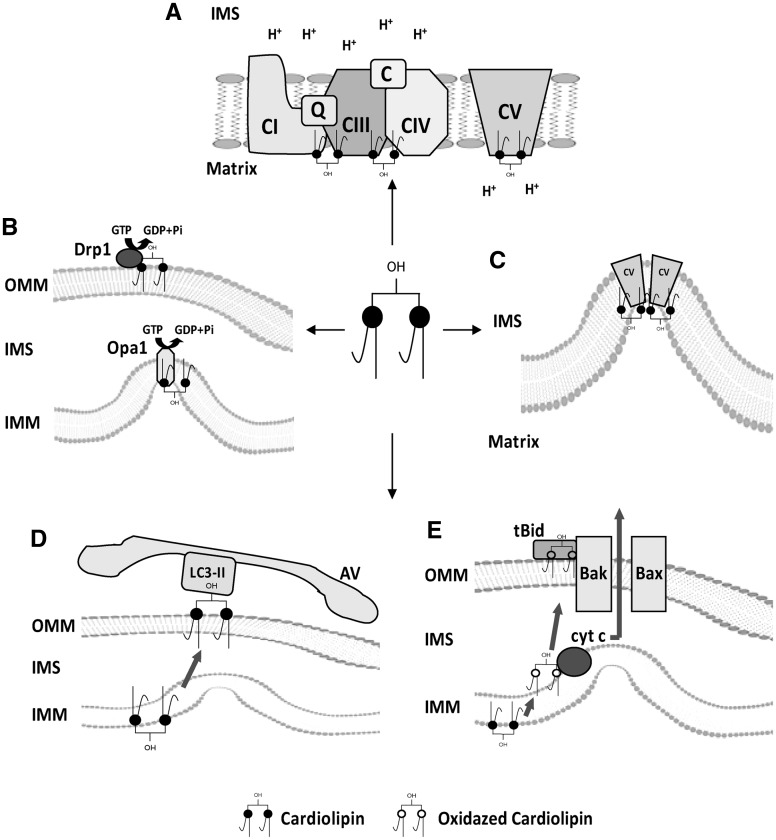
Cartoon representing cardiolipin functions in mitochondria. (**A**) Cardiolipin (CL) is essential for normal electron transport and proton translocation activity of complex I, III and IV and, with its negative charged head group, CL attracts and provides a local pool of protons necessary for complex V function. CL also promotes the assembling of OXPHOS complexes into supercomplexes, which implies an improvement of electron transfer and a reduction in ROS production. (**B**) CL is involved in mitochondrial dynamics. It is critical for the fusion of the IMMs via its interaction with optic atrophy (Opa1), promoting its dimerization and enhancing its GTPase activity. CL also has a role in the fission pathway. After its transfer from the IMM to the OMM, it mediates Drp1 recruitment to mitochondrial membrane surface and enhances Drp1 GTPase activity. (**C**) In concert with complex V, CL promotes cristae formation. CL structure composed of two phosphatidyl moieties with a single glycerol group promotes negative curvature of membrane. (**D**) CL externalization from IMM to OMM acts as an ‘eat-me-signal’ for the autophagic machinery, promoting mitophagy. The negative charged head group of externalized CL interacts with the basic CL binding sites of LC3, which mediates both autophagosome formation and cargo recognition. LC3 recognizes CL more effectively than its metabolites and oxidized CL, suggesting that CL oxidation is not a prerequisite for mitochondria elimination through mitophagy. (**E**) CL plays a role in apoptosis. During apoptosis initiation, CL undergoes peroxidation catalyzed by cytochrome *c*. Mitochondrial injuries generate ROS, which cause a significant amount of CL to flip to the outer leaflet of IMM, where it binds to cytochrome c. After peroxidation and externalization, CL binds a set of apoptotic proteins such as caspase-8, which are recruited to the mitochondrial surface. Caspase-8 cleaves Bid to a truncated form (tBid), which induces Bax/Bak oligometization thereby permeabilizing the membrane and releasing cytochrome *c*. AV, autophagic vacuole; cyt c, cytochrome *c*; IMS, intermembrane space; IMM, inner mitochondrial membrane; OMM, outer mitochondrial membrane.

Mitophagy can result from defects in membrane lipid biosynthetic pathways (39). We have recently shown that muscles, such as quadriceps, are characterized by major lipid alterations in SBMA knock-in mice (22). To determine whether the lipid composition of mitochondria in the muscle of SBMA patients is altered, we performed untargeted lipidomic analysis by mass spectrometry on mitochondria isolated from the quadriceps muscles of SBMA patients and control subjects (Fig. 8A). In SBMA mitochondria, the peculiar structural phospholipid of the inner mitochondrial membrane (IMM), cardiolipin (CL), was decreased by 52%, whereas the levels of phosphatidylethanolamine (PE) and PE-precursor-phosphatidylserine (PS) were increased by 1.5- and 2-fold, respectively, compared with control muscles. To function optimally, immature CL must have its fatty acids remodeled into a mature form (40). We found that 97% of total CL species are represented by molecules having acyl moieties combinations between 3(C18:2)/1(C18:3) and 1(C18:2)/3(C18:1), including tetralinoleoyl cardiolipin (4(C18:2)) which alone accounts for 50% of the total CL (Fig. 8B). In mammals, tetralinoleoyl CL is the most abundant molecular species of CL in highly oxidative tissues, such as skeletal muscle (41). In SBMA muscle mitochondria the ratio between the single CL molecular species to total CL was similar to control, indicating a homogeneous reduction of all the CL molecular species (Fig. 8B). These data suggested defects in synthesis rather than processing of CL to the mature forms. CL is synthesized by cardiolipin synthase (CRLS1), a key enzyme involved in the *de novo* biosynthesis of immature CL (40). We hypothesized that the decreased synthesis in cardiolipin was owing to reduced expression of *CRLS1*. By real-time PCR, we found that the transcript levels of *CRLS1* were decreased by 51% in SBMA muscles compared with control muscles (Fig. 8C). In conclusion, these observations show major morphological and biochemical mitochondrial alterations in the muscle of SBMA patients associated with reduced CL synthesis. Because CL plays a critical role in mitochondrial homeostasis (Fig. 9), our results suggest a causative link between alterations in CL levels, mitochondrial dysfunction and muscle atrophy in SBMA.

## Discussion

Analysis of muscle biopsy specimens revealed that SBMA patients have both neurogenic atrophy and myopathic changes, as previously reported (2,19,20). In addition, SBMA muscle was characterized by the presence of hypertrophic fibers, which are not often detected in the muscle of patients suffering from ALS and denervative diseases, indicating that the presence of a large number of hypertrophic fibers is a feature of SBMA skeletal muscle pathology. Hypertrophic fibers can be detected also at late stage of disease in mice overexpressing polyglutamine-expanded AR (21). On the other hand, such hypertrophic fibers are absent in the muscles of knock-in SBMA mice (22). Interestingly, the size of oxidative hypertrophic fibers was bigger than that of glycolytic fibers, indicating that the hypertrophy of the oxidative fibers exceeds that of glycolytic fibers. We have previously shown that in SBMA patients and knock-in mice muscles composed of both glycolytic and oxidative fibers undergo a metabolic switch toward oxidative phosphorylation (22). Moreover, glycolytic fibers were more severely affected than oxidative fibers, and this was associated with impaired glycolysis (22). It is possible that glycolytic fibers are particularly sensitive in SBMA, and that a compensatory mechanism leads to hypertrophy of oxidative fibers.

Expression of polyglutamine-expanded AR in muscle is key to neurodegeneration (16,17,21). Here, we analyzed for the first time polyglutamine-expanded AR accumulation and subcellular localization in the intact muscle of SBMA patients. We found a significant reduction of polyglutamine-expanded AR in total lysates of SBMA muscles compared with control muscles. This reduction in mutant AR accumulation was not associated with reduced *AR* gene transcription, suggesting additional mechanisms responsible for reduced AR accumulation, such as increased degradation. Polyglutamine-expanded AR is mainly degraded by the ubiquitin proteasome system and autophagic processes (11,42–46). Basal autophagy is increased in animal models of SBMA (22,31,32), and in the muscle of SBMA patients, as shown here. These data suggest that activation of the autophagic/lysosomal pathway may result in increased AR turnover and could be a tissue-specific protective strategy to cope with the expression of mutant AR. Despite the levels of total AR being decreased in SBMA muscle, its accumulation in the nuclear fraction was increased compared with control specimens. Nuclear localization of polyglutamine-expanded proteins plays a critical role in the neurodegenerative process. Huntingtin predominantly localizes to the cytosol, but it translocates to the nucleus upon polyglutamine expansion (47). Moreover, deposition of polyglutamine-expanded proteins in forms of nuclear inclusions has been observed in SBMA, as well as HD, DRPLA and several types of SCAs (48). Importantly, nuclear localization of proteins linked to polyglutamine diseases is a prerequisite to toxicity (10,42,49–51). Consistent with these observations, accumulation of polyglutamine-expanded AR was increased in the nucleus of differentiated SBMA myotubes, but not in proliferating SBMA myoblasts, suggesting that the process of muscle differentiation is associated with an abnormal nuclear accumulation of the disease protein (24). AR nuclear translocation occurs upon androgen binding and is mediated by importin-α and β, which move the cargo import complex through the nuclear pore complex into the nucleus (52). Abnormalities in the nuclear pore complex have recently been described in ALS (53,54), and these defects may contribute to the abnormal subcellular localization of specific proteins linked to motor neuron diseases, such as fused in sarcoma (FUS), TAR DNA-binding protein 43 (TDP-43) and AR. Further analysis is required to address why polyglutamine-expanded AR accumulates in the nucleus, whether it results from increased transport to or reduced export from the nucleus, and whether the nuclear pore complex is dysfunctional in SBMA muscle. Another important aspect consequent to the aberrant nuclear accumulation of polyglutamine-expanded AR is that nuclear AR works as a transcription factor activated by androgens. Several genes regulated by AR and whose expression is altered in SBMA muscle code for mitochondrial proteins (22,55), raising the possibility that an increased accumulation of mutant AR in the nucleus may cause mitochondrial abnormalities by altering the expression of nuclear genes encoding mitochondrial proteins.

Expression of polyglutamine-expanded AR alters mitochondrial homeostasis (reviewed by 56). Mitochondrial abnormalities, including reduced number, mass and membrane potential, were previously reported in cell and animal models of SBMA (22,25). However, a detailed characterization of mitochondria in intact muscle tissues derived from SBMA patients was still missing. Here, we describe for the first time mitochondrial abnormalities detected in muscle biopsy specimens derived from SBMA patients. We found reduced number and altered morphology and distribution of mitochondria in SBMA muscle. Polyglutamine-expanded AR may alter mitochondrial homeostasis through an indirect effect, by modifying the expression of nuclear genes encoding mitochondrial proteins, such as genes involved in glycolysis (22,55). On the other hand, polyglutamine-expanded AR may alter mitochondrial homeostasis and function through a direct effect. Indeed, polyglutamine-expanded AR has been detected in the mitochondrial fraction in motor neuron-derived cells (25), myoblast cells (57) and quadriceps muscle of SBMA patients, as reported here. Interestingly, normal and polyglutamine-expanded AR have been shown to interact with COXVb (58). Polyglutamine-expanded AR may affect mtDNA. Multiple mtDNA deletions were detected in a SBMA patient (59), and mtDNA was reported to be decreased in leucocytes derived from SBMA patients, which negatively correlated with the length of the AR pathogenic polyglutamine tract (60). However, mtDNA was also decreased in female carriers, which are usually non-symptomatic. Here, we report for the first time that mtDNA is decreased in the muscle of SBMA patients. Possibly, this decrease reflects the reduction in the number of mitochondria observed in the muscle of SBMA patients, which is consistent with previous observations in cell models of SBMA (25).

Emerging evidence indicates that autophagy is aberrant in SBMA with tissue-specific changes in autophagy flux and activation. Autophagy flux is blocked in the motor neurons of mice overexpressing polyglutamine-expanded AR (61). On the other hand, autophagy is induced specifically in the muscle of SBMA knock-in and transgenic mice (22,32,61), and SBMA patients, as shown here. The observations that reduced autophagic activity by either Beclin-1 haploinsufficiency or a high-fat diet in SBMA knock-in mice increased skeletal muscle fiber size and significantly extended lifespan suggest that excessive autophagy activation in SBMA muscle is detrimental (22,46). Extending these findings, we describe for the first time to our knowledge that a selective type of autophagy, namely mitophagy, is induced in SBMA muscle. Mitophagy specifically removes mitochondria, a process that occurs even under nutrient-rich conditions and that is mechanistically distinct from basal autophagy (29,38). Mitophagy leads to clearance of damaged mitochondria. In this model, fission events produce two functionally distinct mitochondria, one with high mitochondrial membrane potential (ΔΨ_m_)_,_ and the other with reduced ΔΨ_m_. The depolarized mitochondria are eliminated by mitophagy (62). The overall process is also controlled by mitochondrial dynamics, as it is associated with mitochondrial fragmentation (63). Consistent with this notion, we found augmented expression of fission proteins, such as Drp1 and hFis1, in mitochondria isolated from SBMA muscle, indicating increased mitochondrial fragmentation, a mechanism to segregate dysfunctional or damaged components of the mitochondrial network. Mitophagy is mainly mediated by two pathways, the PINK1/PARK2 and BNIP3/NIX systems (64). In the PINK1–PARK2 pathway mitophagy is usually triggered by an accumulation of PINK1 on the outer mitochondrial membrane (OMM) of dysfunctional mitochondria, where it phosphorylates and activates the E3 ubiquitin ligase PARK2 (37,38). PARK2 then ubiquitinates mitochondrial proteins of the OMM, leading to recruitment of autophagy receptors, such as p62, which recognize the ubiquitinated mitochondria and recruit them to the autophagosome by binding to the lipidated form of LC3. Recently, an alternative pathway for PINK1-mediated mitophagy has been described, which is independent of p62. PINK1 has been shown to directly recruit the autophagy receptors, NDP52 and optineurin, to induce mitophagy in a process that is amplified by PARK2 (65). We found increased association of PINK1 with enhanced ubiquitination of mitochondria isolated from SBMA muscles, indicating that mitophagy is activated in the muscle of SBMA patients. However, we did not observe enhanced recruitment of PARK2 to SBMA mitochondria. Other E3 ubiquitin ligases may regulate mitophagy in SBMA muscle. MUL1 (35) and Gp78 (36) have been described as distinct cellular E3 ubiquitin ligases that eliminate damaged mitochondria, acting in parallel to the PINK1–PARK2 pathway. Although the expression of MUL1 and Gp78 did not change in SBMA muscles compared with control specimens, it is possible that these E3 ubiquin ligases play a role in mitophagy in SBMA muscle. Alternatively, PINK1 can exert functions on SBMA mitochondria other than mediating mitophagy. Further studies are required to clarify the mechanism leading to PINK1 enrichment on SBMA mitochondria and to establish which E3 ubiquitin ligases are responsible for increased mitophagy in SBMA muscle. The BNIP3/NIX system works by directly binding LC3 and docking the nascent autophagosome to the mitochondria with enriched BNIP3 or NIX (64). In muscle, mitochondrial priming is mediated prevalently by the mitophagic protein BNIP3, and to a minor extent by the PINK1–PARK2 signaling pathway (66). Additionally, BNIP3 recruitment, mediated by forkhead box O transcription factors (FOXO), is sufficient to induce mitophagy (67,68). As observed in the present study, SBMA muscle mitochondria had 3-fold-increase in the levels of BNIP3, supporting a role for this mitophagy pathway in SBMA muscle.

Here, we show that autophagy and mitophagy are activated in SBMA muscle, supporting the idea that mitochondrial dysfunction and mitophagy activation are key processes occurring in the muscle of SBMA patients. Mitophagy has been involved in several neurodegenerative diseases, such as Parkinson’s disease and Alzheimer’s disease. Moreover, mitophagy has recently been shown to occur in neuroblastoma cells expressing ALS-linked mutant TDP-43 (69). Pathological conditions associated with the loss of muscle mass and force are often characterized by alterations in the mitochondrial network, function and mitophagy (37). Optineurin and valosin-containing protein are necessary for mitochondrial clearance in response to depolarization, a function that is disrupted by mutations that cause ALS and multisystem proteinopathy (70,71). Mitochondrial dysfunction and autophagy defects are detected in diseases with a primary muscle component, such as Ullrich congenital muscular dystrophy, Bethlem myopathy and congenital myosclerosis, which are caused by mutations in the genes encoding the extracellular matrix protein collagen VI (72). Congenital muscular dystrophy with mitochondrial structural abnormalities (CMDmt) is caused by loss of function mutations in the gene coding for choline kinase β, a key enzyme in the *de novo* synthesis of phosphatidylcholine (PC).This is characterized by the presence of enlarged mitochondria that localize at the periphery of muscle fibers, with central areas devoid of mitochondria (39,73). Muscles of either CMDmt patients, or mice carrying loss of function mutations of choline kinase β, show decreased levels of PC, increased ROS production, decreased complex III activity, increased mitochondrial polyubiquitination and association of PINK1/Parkin, p62, LC3, and decreased mtDNA, suggesting increased mitophagy. Importantly, several of these abnormalities were also present in SBMA muscle. Therefore, maintenance of a functional mitochondrial network is particularly important for skeletal muscle, a post-mitotic tissue that cannot dilute damaged or dysfunctional mitochondria through cellular division, but that is yet able to activate the mitochondria quality control pathway. In physiological conditions, muscle has a highly interconnected mitochondrial network and the entire mitochondrial compartment works as a single dynamic unit to maximize ATP synthesis, which is necessary to support the high ATP demand during contraction (74). Indeed, a highly fused mitochondrial network is important for proper mitochondrial calcium buffering and for the optimal production of ATP, with a higher cristae density and an ideal organization of electron transfer chain components in supercomplexes (75,76). Mitochondrial fusion is advantageous under conditions of high energy demand and optimizes mitochondrial function in stress conditions, as frequently occurs in skeletal muscle (76). Under stress conditions, such as upon expression of polyglutamine-expanded AR, dysfunctional mitochondria are separated from the mitochondrial network and removed via mitophagy, and the occurrence of normal mitochondrial biogenesis may not be sufficient to compensate for this increase in mitochondrial disposal. The question remains as to whether mitophagy is an adaptive process to remove dysfunctional mitochondria and reduce muscle damage, or maladaptive, leading to excessive mitochondrial clearance and unbalanced bioenergetics.

The muscle phenotype of SBMA and CMDmt patients suggests that alterations of the biosynthetic pathways of phospholipids in mitochondria are critical for mitochondrial and skeletal muscle homeostasis and may lead to mitophagy. Mitochondrial membranes have a very well defined lipid composition, with a high content of phospholipids and a low content of sterols and sphingolipids. Most of the mitochondrial lipids are synthesized in the endoplasmic reticulum and then transported to the mitochondria, where they are redistributed between the outer and IMMs. An exception is represented by the diglycerophospholipid CL, which is synthesized in mitochondria from endoplasmic reticulum-derived phosphatidic acid, and is then enriched in the IMM (77). Among phospholipids CL has a peculiar and unique structure composed of two phosphatidyl residues linked by a glycerol moiety (78). CL is involved in numerous distinct mitochondrial activities, it plays important roles in mitochondrial functions, and maintenance of normal CL levels is key to mitochondrial function and stability (Fig. 9). CL controls mitochondrial cristae architecture and the stabilization of physical properties of mitochondrial membranes, and it regulates the stability and function of mitochondrial proteins, including the electron transport chain complexes (79,80). CL binds to and optimizes the activity of complexes III, IV and V, and it regulates mitochondrial dynamics, protein import and apoptosis. Loss of CL decreases the stability and function of complexes III and IV (81), and it results in mitochondrial depolarization and altered mitochondrial function (82). CL also participates in the structural organization and stabilization of the respiratory chain complexes into supercomplexes (83–85). Moreover, redistribution of CL to the OMM has been shown to serve as a signal for mitophagy (86). Cytochrome *c*-catalyzed peroxidation of CL and its externalization occurs during apoptosis initiation (80). Thereafter, CL translocates to the outer leaflet of the OMM, where it interacts with and activates several apoptotic proteins (87). In SBMA muscle, we found decreased levels of all the different CL species. The decrease in CL levels were coupled with increased levels of PE and PS. PE has previously been shown to compensate for some defects concerning mitochondrial morphology owing to the loss of CL in yeast (88). However, CL and PE have distinct effects on the stability and assembly of respiratory complexes, and therefore the increase in PE levels may not be sufficient to compensate for the loss of CL in SBMA muscle (89). Moreover, alterations in CL abundance are associated with pathological states, including aging (90) and diabetes (91). SBMA is an age-related disease often associated with diabetes. Our observations link altered mitochondrial lipid metabolism, perturbation of CL levels, and mitochondrial dysfunction to skeletal muscle atrophy in SBMA. Further investigation is needed to establish whether there is a causative link between muscle hypertrophy, mitochondrial abnormalities, mitophagy and CL loss in SBMA muscle.

In eukaryotic mitochondria, CL is synthesized by CRLS1, a key enzyme involved in the biosynthesis of immature CL. It catalyzes the irreversible condensation reaction in which the phosphatidyl group of cytidine diphosphate diacylglycerol (CDP-DAG) is linked to phosphatidylglycerol (PG) (77). After biosynthesis, immature CL undergoes deacylation and remodeling, to generate the different molecular species with diverse fatty acid composition. In SBMA muscle, CRLS1 expression was decreased by 52%, which may explain why CL is reduced. *CRLS1* is regulated by androgens (92,93), raising the possibility that AR directly regulates its expression. In models of chronic denervation, CL levels are decreased in muscle (94,95) in association with an upregulation of both biosynthesis enzymes CRLS1 and CTP:PA-cytidylyltransferase-1 (95). This compensatory response during chronic muscle denervation differs from our results of CRLS1 downregulation in SBMA muscle. It is possible that, in addition to the effect of denervation, the underlying mechanisms of CL reduction in SBMA muscle may be caused by altered androgen signaling and AR function. The unbalanced phospholipid membrane composition of SBMA mitochondria may induce increased fragility, and it may lower the stress threshold. SBMA mitochondria may be more sensitive to the normal increase in ROS production that occurs during normal skeletal muscle activity, causing transient physiological oxidative stress (37). SBMA muscle mitochondria have an altered membrane lipid composition, which may cause a latent fragility and explain the mitochondriapenia as a result of an imbalance in mitochondrial turnover that favors mitochondrial removal.

In conclusion, we show here for the first time that mitochondrial mass is reduced, mitochondrial morphology and lipid composition is aberrant, and mitophagy is enhanced in the muscle of SBMA patients. These observations have been obtained in muscle biopsy specimens derived from SBMA patients with various degrees of motor impairment and/or disease duration, ranging from a mild to severe phenotype. We propose a model whereby abnormal mitochondria are subjected to quality control and eliminated, leading to aberrant mitochondrial network and function in SBMA muscle. These findings highlight the relevance of mitochondria in disease pathogenesis and identify mitochondrial homeostasis as a target for therapeutic manipulation.

## Materials and Methods

### Human samples

Anonymized control and patient biopsy sample collection was approved by the local Ethics Committee. Written informed consent was obtained from each patient. In all cases, biopsied muscles were clinically affected and showed weakness and/or atrophy. Myopathic changes together with neurogenic atrophy were observed in all muscle biopsies. We studied 19 SBMA patients followed at our Neuromuscular Clinic of the University of Padova (Supplementary Material, Table S1). Main clinical data, including age at disease onset, age at biopsy and first clinical symptoms were recorded for each patient. Genomic DNA was extracted from the peripheral blood according to standard procedures. CAG repeats fragment sizing in *AR* gene was performed on an ABI PRISM 3700 DNA Sequencer (Applied Biosystems, Foster City, CA, USA). The specific length of CAG repeats was further verified via Sanger sequencing. Muscle biopsies were obtained using an open biopsy procedure with the collection of 100–200 mg of muscle tissue. All biopsies were immediately frozen in liquid nitrogen for histopathology and biochemical analyses and stored at −80 °C until analyzed. Muscle biopsies from 18 age- and sex-matched healthy subjects, with no signs of neuromuscular diseases and with normal creatine kinase levels were used as controls. Muscle biopsies from 5 age-matched female controls and 46 age-matched male patients affected with neurogenic diseases, including ALS (32) and sensory-motor neuropathies (14), followed at our Neuromuscular Clinic of the University of Padova, were used for atrophy and HIs evaluation.

### Nuclear/cytosolic and mitochondria fractionation

Nuclear and cytosolic fractions were obtained by treating 50 × 20 μm thick fresh-frozen sections of muscle biopsies with the NE—PER Nuclear and Cytoplasmic Extraction kit (Thermo Scientific), supplemented with protease inhibitors cocktail (Sigma). Protein concentration was determined by BCA assay (Thermo Scientific). For each sample, 30 μg of nuclear and cytosolic proteins were loaded for the different analyses. Nuclear enrichment was tested by western blotting as the ratio between PARP (nuclear marker) and β-tubulin (cytosolic marker) in total lysate, nuclear and cytosolic fractions of each muscle sample. As shown in Supplementary Material, Figure S3, in total lysate and the cytosolic fraction the PARP:β-tubulin ratio was 0.07 ± 0.01 and 0.21 ± 0.14, respectively, whereas in the nuclear fraction this ratio was 6.03 ± 0.70. This procedure yielded a 29- and 86-fold enrichment of the nuclear fraction when compared with cytosolic fraction and the total homogenate, respectively.

Human muscle mitochondria were isolated from frozen muscle specimens (50–100 mg), as previously described (96). Each muscle sample was diluted 1:10 in Buffer A (20 mM HEPES, 100 mMKCl, 1 mM EDTA, 2 mM β-mercaptoethanol, 0.3% BSA), homogenized using a glass pestle in a glass potter and centrifuged at 800*g* for 10 min at 4 °C. The resulting supernatant (S1) was transferred to a new tube and the pellet was diluted 1:20 in Buffer A and centrifuged at 800*g* for 10 min at 4 °C, to increase the yield of mitochondria. The resulting supernatants (S1 + S2) were pooled and centrifuged at 10 000*g* for 10 min at 4 °C. Following this centrifugation step, supernatant was discarded and the pellets containing the mitochondria were suspended in the isotonic MSEM buffer (3 ml/initial g of tissue, 220 mM Mannitol, 70 mM sucrose, 1 mM EDTA, 2 mM β-mercaptoethanol, 5 mM MOPS), aliquoted in several tubes, centrifuged at 20 000*g* for 10 min at 4 °C and stored at −80 °C until further analysis. Mitochondrial enrichment was tested by western blotting as the ratio between TOM20 (mitochondrial marker) and β-tubulin (cytosolic marker) in the total lysate and in the isolated mitochondria of muscle from two SBMA and two control subjects. As shown in Supplementary Material, Figure S4, the total lysate TOM20:β-tubulin ratio was 0.50 ± 0.06, whereas in isolated mitochondria it was 7.85 ± 1.36. This procedure yielded a 16-fold enrichment of the mitochondrial fraction compared with the total homogenate.

### Molecular analyses

To measure mtDNA copy number, total DNA was isolated from muscle tissue using the DNeasy Blood & Tissue Kit (Qiagen). Quantitative polymerase chain reaction was performed using a ABI PRISM 7000 light cycler, using Platinum quantitative PCR SuperMix-UDG with ROX (Invitrogen). The mtDNA copy number was estimated as previously described (97). Briefly, the mitochondrial encoded gene, cytochrome *c* oxidase (COII), was amplified and compared with the amplification profile of the nuclear single copy gene, amyloid precursor protein (APP). The relative level for each gene was calculated using the ‘½^Ct method’. In all experiments, each sample was analyzed in triplicate. Probes were labeled with FAM and TAMRA. For mRNA quantification, total RNA was isolated from muscle biopsies using TRIzol Reagent (Life Technologies). First-Strand cDNA synthesis was performed using High-Capacity cDNA Reverse Transcription Kit (Life Technologies) and transcript levels were quantified by SYBER Green Real-Time PCR (Life Technologies) using the ABI PRISM 7000 sequence detection system. Primer sequences are listed in Supplementary Material, Table S2.

### Biochemical analyses

For western blotting analysis, total muscle lysates were obtained by cutting 30 × 20 μm-thick fresh-frozen sections from each muscle biopsy and placing these sections on ice for 30 min in 200 μl of RIPA buffer (65 mM Tris, 150 mM NaCl, 1% NP-40, 0.25% Na-DOC, 1 mM EDTA, pH 7.4) that contained 2 μl of a protease inhibitor cocktail (Sigma) (24). After centrifugation at 20 000*g* for 20 min at 4 °C, the supernatant was collected and stored at –80 °C until use. Protein concentration was determined by BCA assay (Thermo Scientific). Thirty µg of protein from each sample were loaded for the different analyses. The protein samples were separated by SDS-PAGE (7.5 or 10% polyacrylamide gels) and transferred to a nitrocellulose membrane (Whatman). Membranes were blocked in 5% (w/v) fat-free milk in 0.02 M Tris/HCl pH 7.5, 137mM NaCl, and 0.1% (v/v) Tween-20 for 1 h at room temperature and then incubated overnight at 4 °C with the primary antibodies diluted in blocking solution. Bound antibody was visualized using an ECL reagent (GE Healthcare). Integrated optical density of each band was calculated with commercial software (Gel-Pro Analyzer 3). The primary antibodies used were: anti-AR polyclonal (Santa Cruz, N-20, sc-816, 1:1000); anti-ACTB monoclonal (Chemicon International, MAB1501, 1:20 000); anti-PARP-1 polyclonal (Santa Cruz, H-250, 1:2000); anti-β-tubulin polyclonal (Santa Cruz, H-235, 1:1000); anti-TOM20 polyclonal (Santa Cruz, Sc-11415, 1:1000); anti-SQSTM1/p62 monoclonal (Sigma, 041M4812 1:2000); anti-LC3 monoclonal (Sigma, L7543, 1:1000); anti-Beclin-1 polyclonal (Cell Signaling, 3738, 1:1000); anti-LAMP-1 monoclonal (DSHB, H4A3, 1:400); anti-BNIP3 polyclonal (Sigma, B7931, 1:1000); anti-PINK1 monoclonal (Cell Signaling, D8G3, 1:1000); anti-PARK2 monoclonal (Santa Cruz, Sc-32282, 1:1000); anti-Ubiquitin monoclonal (Millipore, MAB1510, 1:1000); anti-DLP1 monoclonal (BD Biosciences, 611112, 1:1000); anti-Fis1 polyclonal (Alexis, ALX-210-907, 1:1000); anti-ATPase polyclonal (home-made), generous gift of Prof. F. Dabbeni-Sala from University of Padua.

For Blue Native PAGE (BN-PAGE), mitochondrial pellets were resuspended at 10 µg/µl in Native Buffer (Invitrogen) with 4% Digitonin (Sigma) for 1 h on ice and centrifuged 20 min at 16 000*g* at 4 °C. The supernatant was collected and 1% G250 sample buffer additive (Invitrogen) was added. Ten micrograms of mitochondrial membrane proteins were loaded onto a 3–12% Bis−Tris gel (NativePAGE™ Novex^®^ Bis-Tris Gel System, Invitrogen), as manufacturers’ instructions, and transferred to Immobilon-P transfer membrane (Millipore). Membranes were blocked in 2% bovine serum albumin in 0.02 M Tris/HCl pH 7.5, 137 mM NaCl, and 0.1% (v/v) Tween-20 for 1 h at room temperature and then incubated for 1 h at room temperature with the primary antibodies diluted in blocking solution. Visualization of the antibody protein complexes was achieved by using enhanced chemiluminescence (LiteAblot-Turbo, Euroclone) and the ChemiDoc™ XRS+ System (Bio-Rad). Densitometry was performed with the Gel-Pro Analyzer 3 software. The primary antibodies used were: anti-MTCO1 monoclonal (Abcam, ab14705, 1:5000), anti-Complex I subunit NDUFB8 monoclonal (Molecular Probes, 459210, 1:5000) and anti-GRP75 polyclonal (Santa Cruz, sc-13967, 1:5000).

OXPHOS activities were tested as previously described (98). Briefly, muscle biopsies (20–30 mg) were homogenized in Sucrose buffer 250 mM (0.121 g Tris, 0.15 g KCl and 0.038 g EGTA in a final volume of 50 ml). On the same day of the experiment, 0.854 g of sucrose was added to 10 ml of this buffer, and then the homogenate was centrifuged at 600*g* for 10 min at 4 °C. The supernatant was transferred into a new tube on ice for respiratory chain complex analysis. CS was quantified as previously described (98). A protein amount corresponding to a CS activity of 2500, 4000 or 5000 nmol min^−^^1^ mg^−^^1^ was loaded for the different analyses. Protein concentration was determined by Bradford assay (Sigma). The enzymatic activities of respiratory chain complexes I–IV were assayed in duplicate or triplicate with a single-wavelength, temperature-controlled spectrophotometer at 37 °C, as previously described (98). The enzymatic activities for each mitochondrial enzyme was calculated as nmol min^−^^1^ mg^−^^1^ of protein and also normalized to the activity of CS, a mitochondrial matrix enzyme, used as a marker of the abundance of mitochondria within a tissue.

### Lipidomic analysis in mitochondria isolated from muscle

For mitochondria lipid extraction, 100 µl aliquots of mitochondrial suspension (containing 10 µg of protein) were used for lipid extraction. Lipids were extracted according to the method of Bligh and Dyer (99) substituting deuterated dichloromethane with chloroform (100). Briefly, 320 μl of methanol (MeOH) was added to each 100 μl sample. Samples were then vortexed for 60 s. Next, 630 μl of DCM was added, the sample was again vortexed for 60 s and 200 μl of water was added to induce phase separation. The samples were then vortexed for 60 s and allowed to equilibrate at room temperature for 10 min before centrifugation at 8000*g* for 10 min at 10 °C. A total of 10 μl of the lower lipid-rich DCM layer was then collected and diluted in 990 μl of acetonitrile (ACN)/DCM/H_2_O (80:15:5, v/v/v) before liquid chromatography–mass spectrometry (LC–MS) analysis. One microliter of sample (equivalent to 200 pg of proteins) was processed for Nano LC–MS analysis. The LC/MS analyses were run on an Agilent (Agilent, Santa Clara, CA, USA) 6520 accurate mass Quadrupole Time-of-Flight (Q-TOF) LC-MS system governed by Agilent MassHunter software (B.05.00 version). The LC system consists of a 1200 series high-performance LC (HPLC) system coupled on-line to MS through a Chip Cube Interface (Agilent Technologies). Each sample (1 µl) was loaded onto a large capacity chip-column filled with 3 µm Merck ZIC-HILIC, integrating a 500 nl capacity trap-column, a 75 µm × 150 mm column, connection capillaries, and a nano-spray emitter. Solvent A was ACN/DCM/MET/H_2_O (60:10:10:20, v/v/v/v), containing 5 mM ammonium formate, while solvent B was ACN/DCM/H_2_O (80:15:5, v/v/v) containing 5 mM ammonium formate. Peptides were separated with a linear gradient of 0–100% of solvent A in 30 min at a flow rate of 0.35 µl min^−^^1^. The LC effluents were introduced into the Q-TOF spectrometer by Agilent Chip Cube Interface that operated in negative mode (Vap = 1700 V) with nitrogen as desolvating gas at 325 °C and 4.0 l/min; fragmentor, skimmer, and octapole were set at 150, 65 and 750 V, respectively. The Q-TOF operates in MS mode at 2 GHz, extend dynamic range with two reference ions (mass accuracy 5 ppm, resolution about 0.05 Da). Mass spectra were acquired in a data-dependent mode: MS/MS spectra of the five most intense ions were acquired for each MS scan in the 140–1700 Da range. Scan speed was set to 3 MS spectra s^−^^1^ and 5 MS/MS spectra s^−^^1^. Lipid identification and quantification was performed, after conversion of the raw data in XML format, by the software LipidXplorer (101).

### Histological analyses

The AI and HI were measured in routine H&E-stained muscle sections (Olympus BX60) from 19 controls (14 males, 5 females), 15 SBMA patients, 32 ALS patients (26 males, 6 females) and 14 patients with sensory-motor neuropathies (9 males, 5 females), according to Dubowitz (23), using ImageJ software. We measured the minor diameter of each fiber (almost 50 fibers for each muscle sample), because this is not altered by oblique sectioning or kinking of the fibers. Considering that normal males have a minor diameter of 40–80 μm (normal females: 30–70 μm), AI and HI were calculated giving different importance to fibers with mild or severe changes in size, using the following equations:

Atrophy index  = (no. of fibers with a minor diameter <10 µm) * 4 + (no. of fibers with a minor diameter of 10–20 µm) * 3 + (no. of fibers with a minor diameter of 20–30 µm) * 2 + (no. of fibers with a minor diameter of 30–40 µm) * 1.

Hypertrophy index  = (no. of fibers with a minor diameter of 80–90 µm) * 1 + (no. of fibers with a minor diameter of 90–100 µm) * 2 + (no. of fibers with a minor diameter of 100–110 µm) * 3 + (no. of fibers with a minor diameter >110 µm) * 4.

These products are divided by the total numbers of fibers and then multiplied by 1000.

For NADH staining, frozen 8 µm cryosections from five controls and six SBMA patients were brought to room temperature and then incubated for 40 min at 37 °C in 25 ml of 0.2 M Tris/HCl buffer pH 7.4, 25 ml of distilled water, 25 mg of nitro-blue tetrazolium (Sigma N-6876) and 20 mg NADH (Sigma N-8129). Percentage of dark blue area for field was calculated in the NADH-stained sections (Olympus BX60) using ImageJ software, by measuring the number of pixels for field with a dark blue staining. Briefly, the used procedure divided the image into objects and background with respect to the threshold set through Image J (background: low intensity pixels-light blue; object: high intensity pixels-dark blue). Then, the number of pixels for field with a value above threshold was measured.

For immunofluorescence (IF) analysis and imaging, 8 µm muscle cryosections were collected on Superfrost slides, fixed with 4% paraformaldehyde (PFA), treated with 0.5% Triton-X-100, blocked in 10% fetal bovine serum in PBS 1X for 30 min and then incubated overnight at 4 °C with primary antibodies, diluted in blocking solution, against rabbit LC3 (Cell Signaling, polyclonal, #2775, 1:200) and mouse ATP5A (Abcam, monoclonal, ab110273, 1:200). Each primary antibody was sequentially incubated separately on the sections. Appropriate secondary fluorescent antibodies (Alexa-Fluor-488, Invitrogen, A11008, 1:200 for anti-LC3 and Alexa-Fluor-647, Invitrogen, A21235, 1:200 for anti-ATP5A) were used for 1 h at room temperature at the end of the respective section. Slides were mounted using Vectashield medium with DAPI stain (Vector) and examined on a confocal microscope (Leica TCS SP5). In the *z*-axis stacks acquired, each image was separated by 0.5 µm along the *z*-axis. Using the program Fiji, the number of object-voxel was analyzed in each green and red slide of the *z*-stack. Considering the autophagosoma diameter is about 1 µm, only the voxels equal to or greater than 1 µm were analyzed. To evaluate the LC3-ATPase colocalization signal, we used the following formula: $\sqrt{(x1-x2{)}^{2}+(y1-y2{)}^{2}+(z1-z2{)}^{2}}$ and was considered a green-red colocalized voxel only when the green voxel (LC3-autophagosoma) was bigger than the red one (ATPase-mitochondria).

For transmission electron microscopy, muscle specimens were fixed in 3.5% glutaraldehyde in 0.1 M sodium-cacodylate buffer (pH 7.4, room temperature) for 2 h followed by buffer rinse and post fixation for 1 h in 2% osmium tetroxide. The specimens were rapidly dehydrated in graded ethanol and acetone, infiltrated with EPON–acetone (1:1) mixture for 2 h, and embedded in EPON (102). Ultrathin sections were stained with uranyl acetate and lead citrate (Reynolds) and examined with a Philips EM400 transmission electron microscope operating at 100 kV.

### Statistical analysis

Data were expressed as mean values ± SEM. Statistical differences of continuous data from two experimental groups were calculated using Student *t* tests. Comparisons of data from more than two groups were performed using a one-way ANOVA followed by a Fisher’s least significant difference post hoc test. We considered a *P*-value <0.05 to be significant.

## Supplementary Material

Supplementary Material is available at *HMG* online.

## Supplementary Material

Supplementary Data
