# Supplementary material for: Increased mitophagy in the skeletal muscle of spinal and bulbar muscular atrophy patients
Source: Hum Mol Genet. 2017 Jan 13;26(6):1087–103. doi: 10.1093/hmg/ddx019 (PMC5409076; doi:10.1093/hmg/ddx019)
Supplement: Supplementary Data [file ddx019_Supp.pdf]

Increased mitophagy in the skeletal muscle of spinal and bulbar muscular atrophy patients

Doriana Borgia[1], Adriana Malena[1], Marco Spinazzi[2], Maria Andrea Desbats[3], Leonardo Salviati[3], Aaron P. Russell[4], Giovanni Miotto[5,6], Laura Tosatto[7], Elena Pegoraro[1], Gianni Sorarù[1], Maria Pennuto[7*], Lodovica Vergani[1*]

[1]Department of Neurosciences, University of Padova, Padova, Italy

[2] VIB Center for the Biology of Disease, KU Leuven Center for Human Genetics, Leuven, Belgium

[3]Clinical Genetics Unit, Department of Woman and Child Health, University of Padova, Padova, Italy, and IRP Città della Speranza, Padova, Italy

[4]Institute for Physical Activity and Nutrition, School of Exercise and Nutrition Sciences, Deakin University, Burwood, Australia

[5] Department of Molecular Medicine, University of Padova, Italy.

[6] Proteomic Center of Padova University, VIMM and Padova University Hospital, Padova, Italy

[7]Dulbecco Telethon Institute, Centre for Integrative Biology, University of Trento, Trento, Italy

* Corresponding authors:

Lodovica Vergani, PhD

Centro Biomedico Pietro D'Abano-via Orus 2, 35129 Padova, Italy
phone +39.049.8216162
fax +39.049.8216163
e-mail: lodovica.vergani@unipd.it

Maria Pennuto, PhD

Dulbecco Telethon Institute Lab of Neurodegenerative Diseases,
Centre for Integrative Biology (CIBIO), University of Trento,
Via Sommarive 9, 38123 Trento, Italy
Phone +39 0461 285215
Fax: +39 0461 283937
email: MPennuto@Dti.Telethon.it

Supplementary Table 1

SBMA patient	Age at onset (years)	Age at biopsy (years)	CAG repeats number
1	50	52	43
2	31	80	41
3	60	54	43
4	50	59	46
5	26	57	45
6	38	61	44
7	41	45	46
8	64	71	43
9	49	57	44
10	45	61	46
11	37	44	49
12	46	55	48
13	27	28	43
14	29	39	46
15	45	54	48
16	49	55	44
17	43	64	41
18	48	51	44
19	34	37	49

ALS patient	Sex	Age at onset (years)	Age at biopsy (years)
1	M	23	23
2	M	46	48
3	M	41	42
4	M	45	46
5	M	55	55
6	M	62	62
7	M	67	67
8	M	81	83
9	M	77	81
10	M	60	61
11	M	77	77
12	M	61	61
13	M	38	39
14	M	38	40
15	M	46	47
16	M	49	50
17	M	53	54
18	M	55	56
19	M	56	57
20	M	55	57
21	M	58	59
22	M	63	64
23	M	64	66
24	M	67	67
25	M	67	68
26	M	73	73
27	F	30	33
28	F	45	48
29	F	51	51
30	F	50	55
31	F	65	65
32	F	64	66

Nerurogenic atrophy	Sex	Age at onset (years)	Age at biopsy (years)
1	M	23	23
2	M	46	48
3	M	41	42
4	M	45	46
5	M	55	55
6	M	62	62
7	M	67	67
8	M	81	83
9	M	77	81
10	M	60	61
11	M	77	77
12	M	61	61
13	M	38	39
14	M	38	40

Control	Sex	Age at biopsy (years)
1	M	52
2	M	42
3	M	55
4	M	53
5	M	63
6	M	36
7	M	45
8	M	28
9	M	42
10	M	32
11	M	45
12	M	45
13	M	44
14	M	79
15	M	38
16	M	38
17	M	19
18	M	47
19	F	27
20	F	38
21	F	39
22	F	44
23	F	50

Table S1. List of muscle biopsy specimens collected from patients affected with SBMA, ALS, and other neurogenic diseases.

Supplementary Table 2

	Forward primers	Reverse primers	Probe
**APP**	5'-TTTTTGTGTGCTCTCCCAGGTCT-3'	5'-TGGTCACTGGTTGGTTGGC-3'	5'-CCCTGAACTGCAGATCACCAATGTGGTAG-3′
**AR**	5'-TTGTCCACCGTGTGTCTTCTTCTGC-3'	5'-TGCACTTCCATCCTTGAGCTTGGC-3'	
**COII**	5'-CGTCTGAACTATCCTGCCCG-3'	5'-TGGTAAGGGAGGGATCGTTG-3'	5'-CGCCCTCCCATCCCTACGCATC-3'
**COX4**	5'-CATGTGGCAGAAGCACTATGTGT-3'	5'-GCCACCCACTCTTTGTCAAAG-3'	
**CRLS1**	5'-CCCAGTTCTGGGCTATTTGA-3'	5'-TCTTTGATTGGCCCAGTTTC-3'	
**ERRα**	5'-TTCTCATCGCTGTCGCTGTCT-3'	5'-CAGCCGCCGCACTAGTTG-3'	
**Gp78**	5'-ACCTCCTGTCCAACATGCAG-3'	5'-GCAATCCGAGACCCATCGAA-3'	
**MFN1**	5'-TGTTTTGGTCGCAAACTCTG-3'	5'-CTGTCTGCGTACGTCTTCCA-3'	
**MFN2**	5'-ATGCATCCCCACTTAAGCAC-3'	5'-CCAGAGGGCAGAACTTTGTC-3'	
**MnSOD**	5'-CTTCAGCCTGCACTGCCGTTCAAT-3'	5'-CTGAAGGTAGTAAGCGTGCTCCC-3'	
**MUL1**	5'-GCTGTTATAGAAGGAGCTGTGC-3'	5'-GCACTGTGTTGGTCCTCTGA-3'	
**NRF1**	5'-GGTGCAGCACCTTTGGAGAA-3'	5'-CCAGAGCAGACTCCAGGTCTTC-3'	
**PGC-1α**	5'-TCAGTCCTCACTGGTGGACA-3'	5'-TGCTTCGTCGTCAAAAACAG-3'	
**PGC-1β**	5'-CTGCTGGCCCAGATACACTGA-3'	5'-ATCCATGGCTTCATACTTGCT-3'	
**RPLPO**	5'-GTGATGTGCAGCTGATCAAGACT-3'	5'-GATGACCAGCCCAAAGGAGA-3'	
**TFAM**	5'-GAACAACTACCCATATTTAAAGCTCA-3'	5'-GAATCAGGAAGTTCCCTCCA-3'	

Table S2. List of primers used for real-time PCR analysis of gene expression.

Supplementary Figure 1

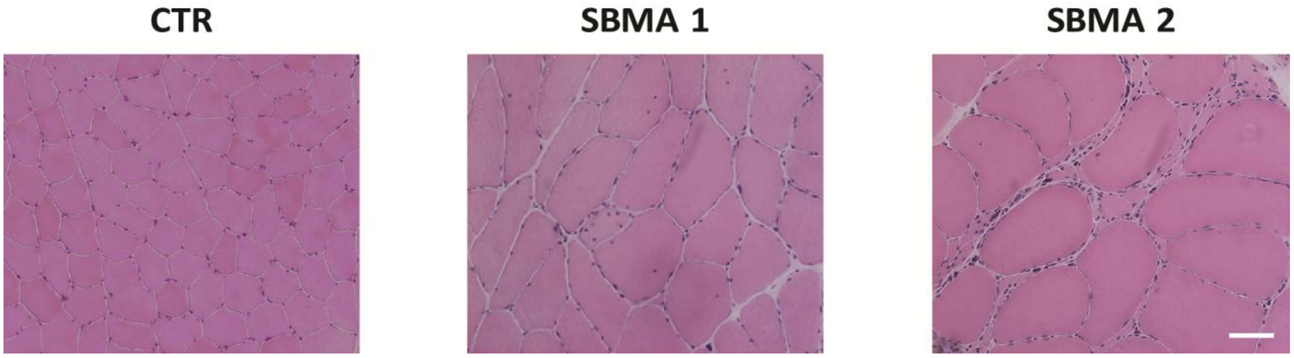

Figure S1. Atrophic and hypertrophic fibers in SBMA muscle.
H&E staining revealed the presence of small atrophic fibers together with enlarged, hypertrophic fibers in the quadriceps muscle of SBMA patients compared to age-matched control specimens. Shown are representative images from 1 control subject and 2 SBMA patients. Scale bar, 80 µm.

Supplementary Figure 2

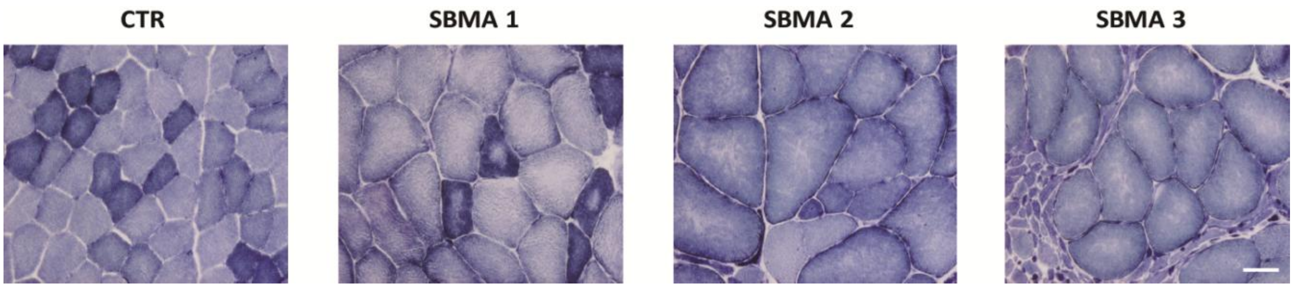

Figure S2. Both oxidative and glycolytic fibers are atrophic and hypertrophic in SBMA muscle.
NADH staining revealed the presence of small atrophic fibers together with hypertrophic glycolytic and oxidative fibers in the quadriceps muscle of SBMA patients compared to age-matched control specimens. Shown are representative images from 1 control subject and 3 SBMA patients. Scale bar, 80 µm.

Supplementary Figure 3

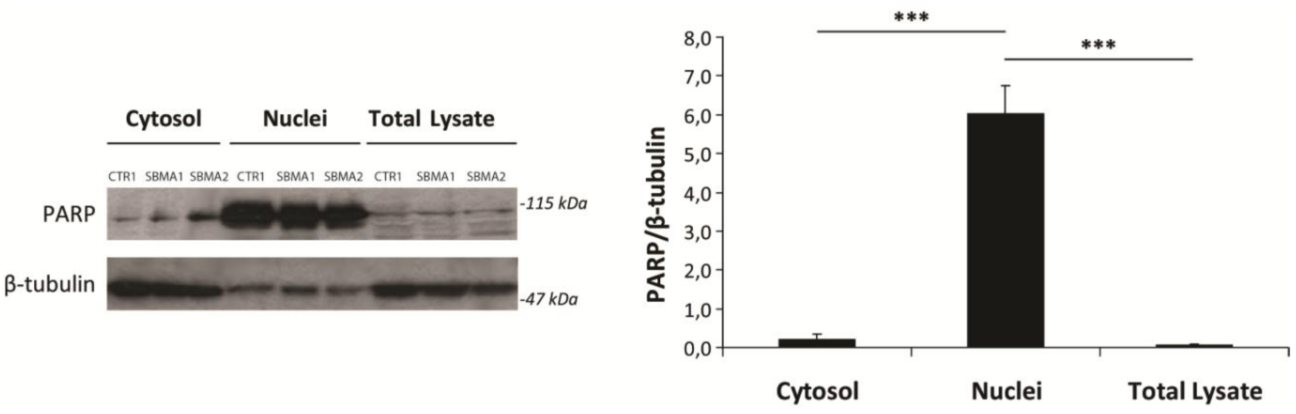

Figure S3. Purity of nuclear extracts.

Western blotting analysis of PARP (nuclear marker) and β-tubulin (cytosolic marker) in cytosolic fraction (cytosol), nuclear fraction (nuclei), and total lysates obtained from SBMA patient and control muscle samples. Nuclear enrichment was evaluated as the ratio between PARP and β-tubulin levels. Graph, mean ± SEM, n = 2 SBMA patients and 1 control (CTR) subject. Significance by Student *t* test: ***$p < 0.001$.

Supplementary Figure 4

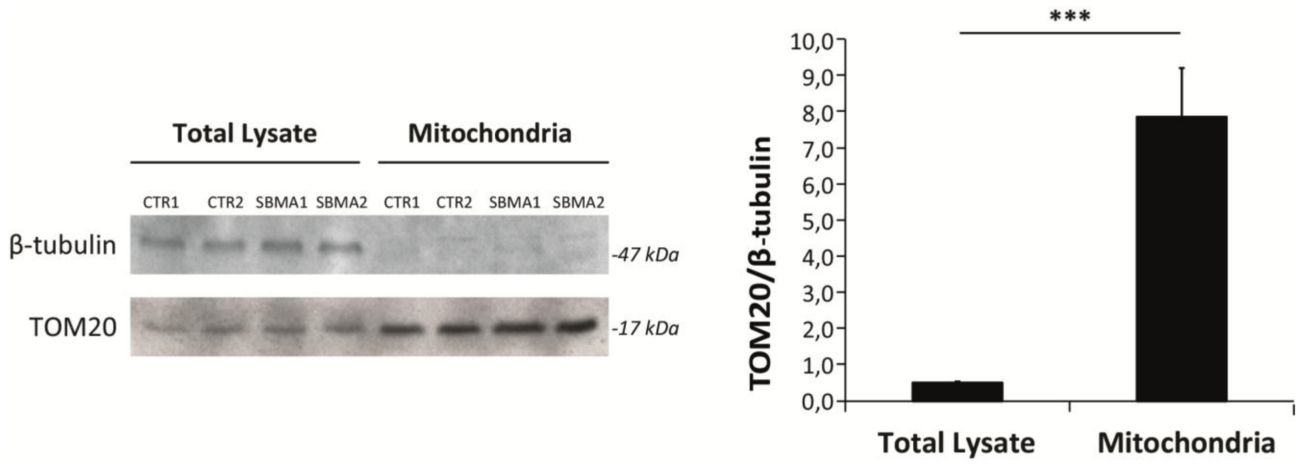

Figure S4. Purity of mitochondrial extract.

Western blotting analysis of TOM20 (mitochondrial marker) and β-tubulin (cytosolic marker) in muscle total lysates and isolated mitochondria derived from SBMA patient and control samples. Mitochondrial purification was evaluated as the ratio between TOM20 and β-tubulin levels. Graph, mean ± SEM, n = 2 SBMA patients and 2 control (CTR) subjects. Significance by Student *t* test: ***$p < 0.001$.

Supplementary Figure 5

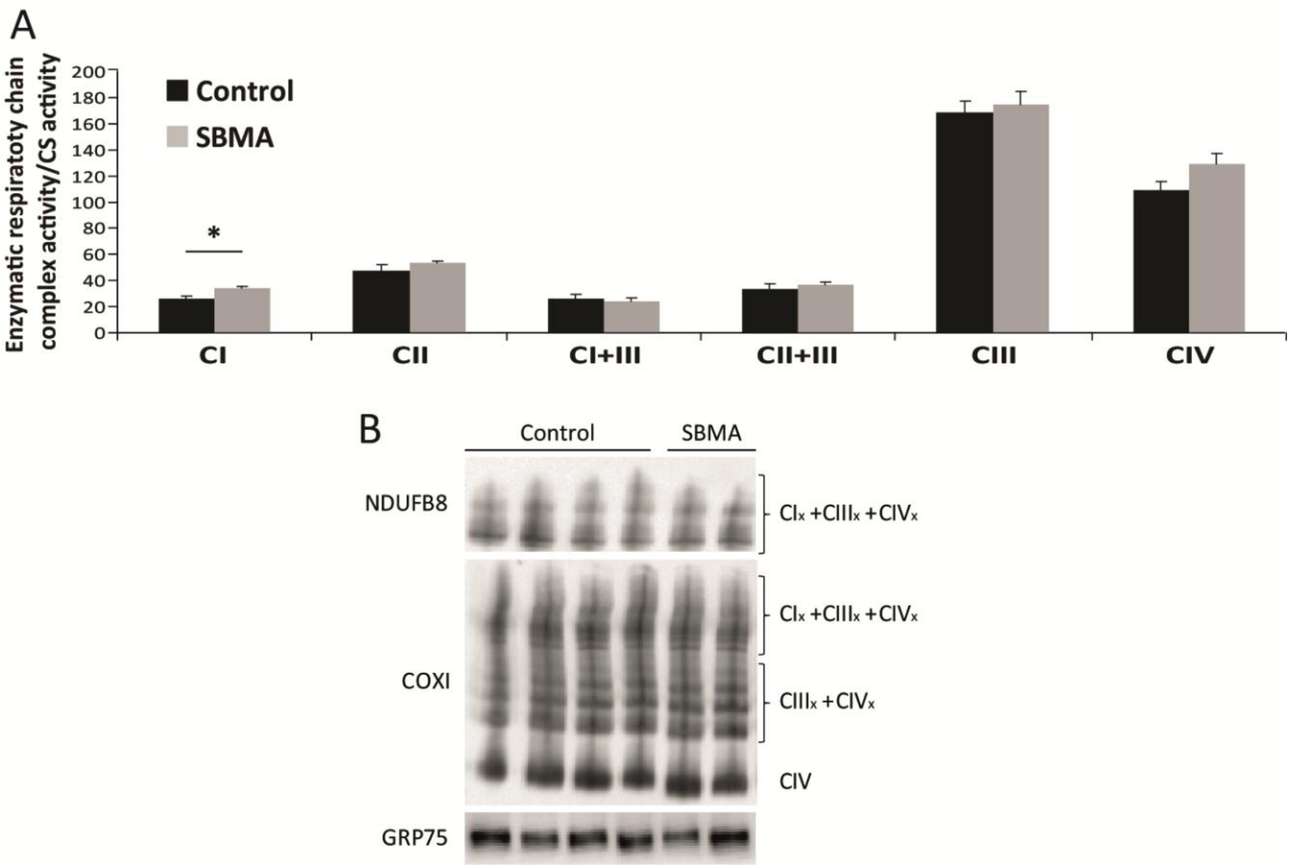

Figure S5. Normal OXPHOS activity upon normalization to CS activity and normal supercomplex expression and assembly in SBMA muscle.

A) Enzymatic activity of respiratory chain complexes I–IV normalized to the CS activity. Graph, mean ± SEM, n = 6 SBMA patients and 4 control subjects. Significance by Student *t* test: $*p < 0.05$.

B) Representative Blue Native PAGE (BN-PAGE) analysis of mitochondrial supercomplexes in isolated muscle mitochondria from 4 control subjects and 4 SBMA patients. Mitochondrial supercomplexes were recognized with antibodies against a subunit of Complex I (NDUFB8) and Complex IV (COXI). GRP75, a marker of mitochondrial matrix, was used as loading control.

Supplementary Figure 6

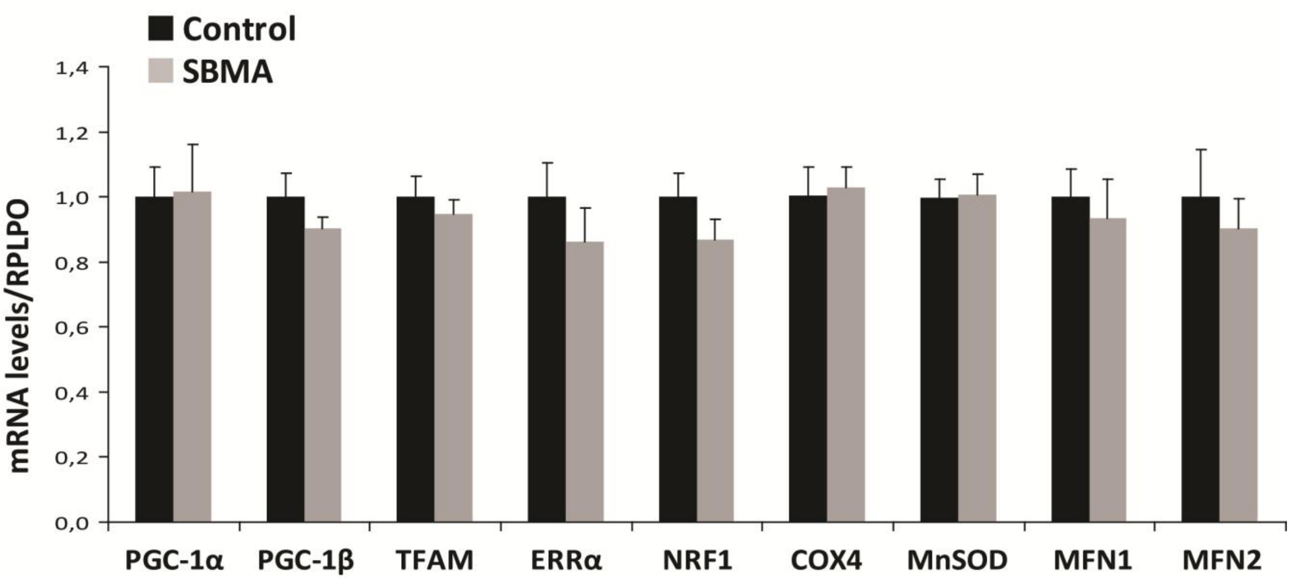

Figure S6. Normal expression of genes involved in mitochondrial biogenesis and function in muscles from SBMA patients. RT-PCR analysis of the transcript levels of genes involved in mitochondrial biogenesis and function, including *peroxisome proliferator-activated receptor-gamma coactivator 1 alpha* and *1 beta* (*PGC-1α* and *PGC-1β*), *mitochondrial transcription factor A* (*TFAM*), *estrogen-related receptor alpha* (*ERRα*), *nuclear respiratory factor 1* (*NRF1*), *cytochrome c oxidase 4* (*COX4*), *manganese-dependent superoxide dismutase* (*MnSOD*) and *mitofusin 1 and 2* (*MFN1* and *MNF2*), normalized to *large ribosomal protein* (*RPLPO*). Graph, mean ± SEM, n = 14 SBMA patients and 18 control subjects.

Supplementary Figure 7

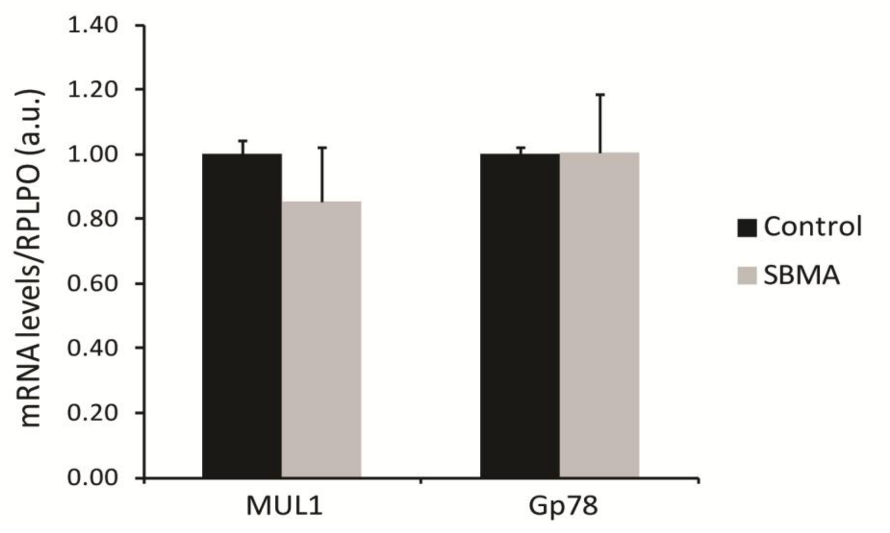

Figure S7. The expression of the E3 ubiquitin ligases, MUL1 and Gp78, is not altered in the muscle of SBMA patients. RT-PCR analysis of the transcript levels of genes involved in mitophagy, including *MUL1 and Gp78*. Graph, mean ± SEM, n = 5 SBMA patients and 3 control subjects.
